# Genome size evolution in the beetle genus *Diabrotica*

**DOI:** 10.1093/g3journal/jkac052

**Published:** 2022-03-02

**Authors:** Dimpal Lata, Brad S Coates, Kimberly K O Walden, Hugh M Robertson, Nicholas J Miller

**Affiliations:** 1 Department of Biological Sciences, Illinois Institute of Technology, Chicago, IL 60616, USA; 2 USDA-ARS, Corn Insects & Crop Genetics Research Unit, Ames, IA 50011, USA; 3 Department of Entomology, University of Illinois at Urbana-Champaign, Urbana, IL 61820, USA

**Keywords:** corn rootworm, MITEs, genome size

## Abstract

Diabrocite corn rootworms are one of the most economically significant pests of maize in the United States and Europe and an emerging model for insect–plant interactions. Genome sizes of several species in the genus *Diabrotica* were estimated using flow cytometry along with that of *Acalymma vittatum* as an outgroup. Genome sizes ranged between 1.56 and 1.64 gigabase pairs and between 2.26 and 2.59 Gb, respectively, for the *Diabrotica* subgroups fucata and virgifera; the *Acalymma vittatum* genome size was around 1.65 Gb. This result indicated that a substantial increase in genome size occurred in the ancestor of the virgifera group. Further analysis of the fucata group and the virgifera group genome sequencing reads indicated that the genome size difference between the *Diabrotica* subgroups could be attributed to a higher content of transposable elements, mostly miniature inverted-transposable elements and gypsy-like long terminal repeat retroelements.

## Introduction 

The family Chrysomelidae is one of the largest families of phytophagous beetles (order: Coleoptera), with nearly 40,000 species. A large number of species are important agricultural and forestry pests causing negative economic impacts (Reid 1995; Nie et al. 2020). A subtribe of Chrysomelidae, Diabroticina includes important agricultural pests from the genera *Acalymma*, *Cerotoma*, and *Diabrotica* (Toepfer et al. 2009). Many species of the genus *Acalymma* are specialists on Cucurbitaceae, with *Acalymma vittatum*, the striped cucumber beetle, being one of the key pests of cucurbits in the northeastern United States (Lewis et al. 1990). The bean leaf beetle, *Cerotoma trifurcata*, is an important pest of leguminous crops such as peas and soybeans throughout the eastern United States (Koch et al. 2004). *Diabrotica*, the most diverse genus (Eben and Espinosa de los Monteros 2013), includes some of the most destructive insect pests impacting US agriculture. *Diabrotica* spp. are divided into 3 groups: signifera, fucata, and virgifera, with the latter 2 containing recognized pest species (Krysan 1986). The species in the fucata group are multivoltine and polyphagous, while species in the virgifera group are univoltine and oligophagous (Branson and Krysan 1981; Krysan 1982). *Diabrotica undecimpunctata* (southern corn rootworm) within the fucata group is a generalist feeder that feeds on several crops, including cucurbits, peanuts, and maize in the southern United States (Jackson et al. 2005). *Diabrotica virgifera* (western corn rootworm), *Diabrotica barberi* (northern corn rootworm), and *Diabrotica virgifera zeae* (Mexican corn rootworm) from the virgifera group are specialist feeders and are pests of maize. *Diabrotica virgifera* is most abundant in the US Corn Belt but is found throughout much of the United States as well as parts of Canada and Mexico. *Diabrotica virgifera* and *Diabrotica barberi* are sympatric in the northern part of the US Corn Belt, while *D. v. zeae* is sympatric with *D. v. virgifera* over part of their range in Texas, Arizona, and Mexico (Bragard et al. 2019).

As the name “corn rootworm” suggests, several *Diabrotica* species cause substantial economic damage to maize agriculture. The western corn rootworm, *D. v. virgifera*, is considered one of the most destructive pests of maize throughout the US Corn Belt in the United States, and it accounts every year for over $1 billion in yield losses and pest management costs (Sappington et al. 2006). The species has also been introduced into Europe and has become widespread because of a combination of transatlantic introductions and intracontinental movement (Ciosi et al. 2008, 2011; Miller et al. 2010). *Diabrotica barberi* is a serious maize pest but is less widespread than *D. v. virgifera* (Capinera 2008). Maize-specialist corn rootworms have proven to be highly adaptable to a variety of pest management tactics. Resistance has evolved to a variety of synthetic insecticides (Ball and Weekman 1962; Meinke et al. 1998; Pereira et al. 2015; Souza et al. 2019, 2020) to most rootworm active transgenic maize varieties (Gassmann et al. 2014; Calles-Torrez et al. 2019) and to cultural control methods (Krysan et al. 1984; Gray et al. 2009). Although *D.* *undecimpunctata* is more widely distributed, it is unable to survive the winter temperatures of the US Corn Belt, and it is considered an occasional pest of maize.

The genus *Diabrotica* is emerging as a model for insect–plant interactions in generalist *vs.* specialist herbivory. The ancestral state for the genus is thought to be generalist feeding with a host plant range that includes Cucurbitaceae, Fabaceae, and Poaceae, which is retained in the fucata group (Eben and Espinosa de los Monteros 2013). Following the split between the fucata and virgifera groups, around 30 million years ago, the virgifera group specialized on Poaceae (Eben and Espinosa de los Monteros 2013). Consequently, pest *Diabrotica* includes both generalist and specialist species that share a common, experimentally tractable host plant in maize.

The benzoxazinoid DIMBOA (2,4-dihydroxy-7-methoxy-1,4-benzoxazin-3-one) is one of the major secondary metabolites produced by maize plants (Sasai et al. 2009). Specialist virgifera group species and generalist fucata group species respond distinctly to this metabolite. *Diabrotica virgifera* larvae gained significantly more dry weight when fed wild-type plants compared to larvae fed mutant plants, deficient for DIMBOA biosynthesis. However, *D. undecimpunctata* performed equally well when fed on both types of plants (Alouw and Miller 2015). The enhanced performance of specialist *D. v. virgifera* may be related to its ability to use DIMBOA as a signal to locate nutritious parts of roots, while the generalist from the fucata group does not (Robert et al. 2012). Further, RNA-Seq studies showed transcripts encoding for a CYP9-like cytochrome P450 monooxygenase were expressed in *D. v. virgifera* larvae feeding on wild-type plants but not in larvae feeding on benzoxazinoid-deficient mutant plants (Miller and Zhao 2015), suggesting a cytochrome P450 mediated adaptation to benzoxazinoids in *D. v. virgifera*.

Given the economic importance of and growing research interest in *Diabrotica* beetles, there has been considerable interest in obtaining sequences of their genomes and understanding genetic mechanisms for adaptations. Most *Diabrotica* genetics and genomics research so far has been concentrated on *D. v. virgifera* (Gray et al. 2009; Miller et al. 2010). The genome of *D. v. virgifera* is one of the larger genomes among beetles and is estimated to be around 2.58 Gb (Coates et al. 2012), whereas the average genome size for Coleoptera is 0.76 Gb (Schoville et al. 2018; Gregory 2021). Increased sizes of eukaryotic genomes are generally attributed to corresponding increased numbers of repetitive DNA elements (Kidwell 2002), where a large proportion of repeats are composed of different transposable element (TE) sequences (Kojima 2020). Eukaryotic transposons are divided into retroelements that propagate by an RNA intermediate (class I) and DNA elements (class II) that mobilize by a “cut-and-paste” mechanism (Finnegan 1989; Wicker et al. 2007).

There is evidence that the *D. v. virgifera* genome contains a high proportion of repetitive elements (Coates et al. 2012, 2014). The *cadherin* gene of *D. v. virgifera* is approximately 13.3-times larger than the *Tribolium castaneum* ortholog due to much larger introns. The presence of numerous MITE-like elements within the *cadherin* gene of *D. v. virgifera* indicates that the difference in the gene size is due to the insertion of TEs in the *D. v. virgifera* introns (Coates et al. 2012). Class I BEL-like long terminal repeat (LTR) retrotransposons have been also found in the *D. v. virgifera* genome (Coates et al. 2014). Initially, MITEs were found as key components of plant genomes, as they are frequently associated with genes with high copy numbers indicating a possible role in gene expression and genome evolution (Santiago et al. 2002; Oki et al. 2008). They are also found in animals, including mosquitoes, *Drosophila*, fish, and humans (Deprá et al. 2012). Similar to MITES, LTR retrotransposons were also first discovered in plants. They are usually located largely in intergenic regions and are often the single largest component of plant genomes (Kumar and Bennetzen 1999; Feschotte et al. 2002). Previous studies have also revealed that both MITES and LTR retrotransposons can modify gene expression by inserting into promoter regions (Butelli et al. 2012; Wang et al. 2017; Liu et al. 2019). TE integration and excision can introduce novel variation (McClintock 1950; Wendel and Wessler 2000). Transposons can cause mutations by inserting themselves into functional regions and causing change by either modifying or eliminating gene expression (Feschotte 2008; Oliver and Greene 2009). They may also lead to genomic rearrangement (Maumus et al. 2015; Mat Razali et al. 2019).

Although the genome size of *D. v. virgifera* has been reported, no genome size data have been obtained for the other species in the genus *Diabrotica* and related genera. Since the genome size of *D. v. virgifera* is relatively large, we hypothesized that there has been a recent expansion in genome size in the lineage leading to it. We tested this hypothesis by estimating and comparing the genome sizes of *D. v. virgifera* with those of several *Diabrotica* species and an outgroup species, *A. vittatum.* As a high proportion of repetitive elements were found in the *cadherin* gene of *D. v. virgifera*, we further hypothesized that genome size expansion in the lineage leading to *D. v. virgifera* was due to a general increase in repetitive elements*.* To test this hypothesis, we looked at the nature and quantity of repetitive elements in the virgifera group and compared it with the fucata group.

## Materials and methods

### Sample collection for flow cytometry

Specimens of *D. v. virgifera* and *A. vittatum* were collected from a maize field in Illinois in 2017, while *D. barberi* were collected from Wisconsin by Tracy Schilder, Wisconsin Department of Agriculture. Specimens of *D. v. zeae* were collected from Texas by Thomas Sappington, US Department of Agriculture Agricultural Research Service and *D. balteata* were provided by Blair Siegfried and Heather McAuslane, University of Florida, from a laboratory colony. *Diabrotica undecimpunctata* were obtained from Crop Characteristics (Farmington, Minnesota, USA). Adult male *Periplaneta americana*, which were used as an external reference (Guo et al. 2015; He et al. 2016) for flow cytometric measurement, were obtained from Carolina Biological Supply (Burlington, North Carolina, USA). All samples were flash-frozen in liquid nitrogen and preserved at −80_**°**_C.

### Sample preparation for flow cytometry

Genome size estimates were generated for 8 individuals from 5 species of *Diabrotica* and 1 species of *Acalymma*. Preparations of nuclei were based on the method of Hare and Johnston (2012). The heads of single individuals were homogenized in 1 ml of cold Galbraith buffer (45 mM MgCl_2_, 30 mM sodium citrate, 20 mM MOPS, 0.1%(v/v) Triton-X-100, pH 7.0, and 1_** µ**_g/ml boiled ribonuclease A) placed in a 7 ml Kontes Dounce. The homogenate was filtered through a 20 μm nylon mesh in a 1.5 ml Eppendorf tube. Nuclei were stained with propidium iodide (PI) at 50 μg/ml in the dark at 4_**°**_C for an hour. In addition to the test sample, the brain tissue of *P. americana* was used as a standard (Hanrahan and Johnston 2011). The brain tissue of *P. americana* was dissected out, and the nuclear suspension was prepared and stained as described above.

### Flow cytometric analysis

Stained nuclei were analyzed using an Attune NxT Flow Cytometer (Thermo Fisher Scientific, Waltham, MA, USA). The propidium iodide-stained nuclei were excited by exposing them to the 488 nm blue laser. Red fluorescence from the propidium iodide was collected using the YL2 detector channel. The calibration of the flow cytometer was performed using a standard manufacturer’s protocol before use. During each sample run, the linearity of the fluorescence measurement was confirmed by checking that the mean channel number of the 4C nuclei (G2 phase) was double that of 2C nuclei (G1 phase). At least 1,000 nuclear events were collected under each unknown and standard 2C peak. The nuclei peak (PI fluorescence histogram) and coefficient of variation (CV) for each peak of interest (sample and standard) were obtained using the gating function in the Attune Software. The CV was less than 5% which is considered appropriate for accurate genome size estimates (Dolezel et al. 2007; Tomaszewska et al. 2021). The known genome size of the external standard (3.34 Gb, Hanrahan and Johnston 2011) and the relative fluorescence obtained from the sample and external standard were then used to estimate the genome size using the following formula:

Sample 1C DNA content = [(sample 2C mean peak position)/ (standards 2C mean peak position)] × standards 1C.

Genome size variations were analyzed using analysis of variance (ANOVA) and Tukey’s honest significant difference (HSD) post hoc analyses using R statistical software (version: 4.10) (R Core Team 2021). Letters were assigned showing significance based on Tukey HSD post hoc test using R statistical software (version: 4.10) (R Core Team 2021).

### Sampling and genomic DNA sequencing

Separate sample collection and preparation were done to obtain the data from Illumina whole-genome shotgun sequencing which were used to analyze the repetitive DNA content of *D. barberi, D.* *undecimpunctata*, and *D. v. virgifera*. Adult *D. barberi* (*n*** **=** **71) and *D. undecimpunctata* (*n*** **=** **50) were collected from maize fields near Ames, Iowa, and Monmouth, Illinois, respectively. Each sample was pooled by species, flash-frozen in liquid nitrogen and ground in liquid nitrogen, and then DNA extracted from ∼3.0 mg of tissue using the Qiagen DNeasy Blood and Tissue Extraction kit, with modifications as described (Coates et al. 2014). Two micrograms of extracted DNA was submitted to the Iowa State University DNA Facility (Ames, IA, USA) from which ∼500 bp insert indexed sequencing libraries were generated using the Illumina TruSeq v2 Library Construction Kit (Illumina, San Diego, CA, USA). Single-end 100-bp reads were generated from *D. barberi* and *D. undecimpunctata* libraries in separate lanes of an Illumina HiSeq2500. Raw reads were submitted to the National Center for Biotechnology Information (NCBI) Short Read Archive (SRA) under accessions SRR13363759 and SRR13364002 for *D. barberi* and *D. undecimpunctata*, respectively.

*Diabrotica virgifera* adult females of inbred line Ped12, developed by the USDA-ARS North Central Agricultural Research Laboratory were used for the genomic DNA isolation. Briefly, whole beetles were homogenized in an SDS-based cell lysis solution followed by overnight incubation with Proteinase K at 55_**°**_C. Cellular debris was pelleted and RNA was digested with RNaseA. The homogenate was mixed with a high-salt solution and incubated overnight at 4_**°**_C. The DNA in the supernatant was precipitated overnight with ethanol at −20_**°**_C. DNA was quantified on an Invitrogen Qubit. A paired-end short-insert genomic DNA library was prepared at the Roy J. Carver Biotechnology Center at the University of Illinois at Urbana-Champaign using an Illumina TruSeq DNAseq Sample Prep kit. Reads were sequenced to 100 bp with the Illumina TruSeq SBS sequencing kit version 3 on an Illumina HiSeq 2000 instrument using Casava 1.8 for basecalling. Raw reads were submitted to the Biotechnology Information (NCBI) Short Read Archive (SRA) under accession SRR6985755.

Sequencing generated 90 million single-end reads of 100 bp for *D. barberi*, 118 million single-end reads of 100 bp for *D.* *undecimpunctata*, and 116 million 100-bp paired-end reads for *D. v. virgifera*.

### Annotation and Quantification of Repeat Content from sequencing data.

Raw reads were quality-filtered using fastp software (version 0.20.1) with a minimum 20 average Phred score. Reads mapping to mitochondrial genome sequences of *Diabrotica* species available through the NCBI website (KF658070.1, KF669870.1) were identified (minimap2 v2.17) and filtered out as implemented in the SSRG workflow (Pombert 2021). Repetitive elements in the genomes of *D.* *undecimpunctata, D. barberi*, and *D. v. virgifera* were assembled and quantified using dnaPipeTE v1.3 (Goubert et al. 2015) and annotated using the DeepTE tool (Yan et al. 2020). To quantify the proportion of TEs, dnaPipeTE uses samples of sequence reads instead of genome assemblies, making this pipeline (dnaPipeTE) applicable for genomes with lower sequencing depth. The pipeline performed assembly of repetitive reads into contigs from low coverage sampling of raw reads using Trinity (Grabherr et al. 2011) and annotated them using RepeatMasker (Smit et al. 1996) with built-in Repbase libraries (Bao et al. 2015, version 2017-01-27). Quantification was done by mapping a random sample of reads onto the assembled repeats. The parameters set as the benchmark for repeat content analysis for genomes greater than 500 Mb (Goubert et al. 2015), including the coverage parameter, were used to run dnaPipeTE. The pipeline was run for all 3 species using 0.1x coverage. In addition, 0.1x coverage was chosen based on the high N50 metric and plateauing point of TEs, i.e. increasing the coverage beyond 0.1x only marginally increased the proportion of TEs for all 3 species. The dnaPipeTE pipeline does not annotate novel repeats that do not match an entry in the included Repbase library. A high proportion of repeats from each of the 3 beetle species were not annotated by dnaPipeTE. DeepTE, a deep learning method based on convolutional neural networks, was used to classify and annotate the unknown TEs. DeepTE uses 8 trained models to classify TEs into superfamilies and orders. All the TE contigs assembled by dnaPipeTE were analyzed using DeepTE, whether or not they had been previously classified by dnaPipeTE. Combining the results of the assembly and quantification by dnaPipeTE with the classification results from DeepTE allowed the abundance of repeat families in the genomes of all 3 species to be determined.

For comparison with the dnaPipeTE de novo assembly of repetitive elements, the percentage of repetitive elements in the *D. v. virgifera* genome assembly (NCBI RefSeq accession GCF_003013835.1) was analyzed with RepeatModeler version 2.0.1 (Flynn et al. 2019). Repeatmodeler is a de novo TE identification package that uses 3 repeat finding programs (RECON, RepeatScout, and LtrHarvest/Ltr_retriever) to discover repetitive DNA sequences in the genome. These repetitive DNA sequences were annotated by repeatClassifier based on the similarity to RepBase and Dfam databases. The annotated library produced was used as input to RepeatMasker to detect and mask repeats in the genome. Default parameters were used to run RepeatModeler.

## Results

### Genome size

**Fig. 1.**
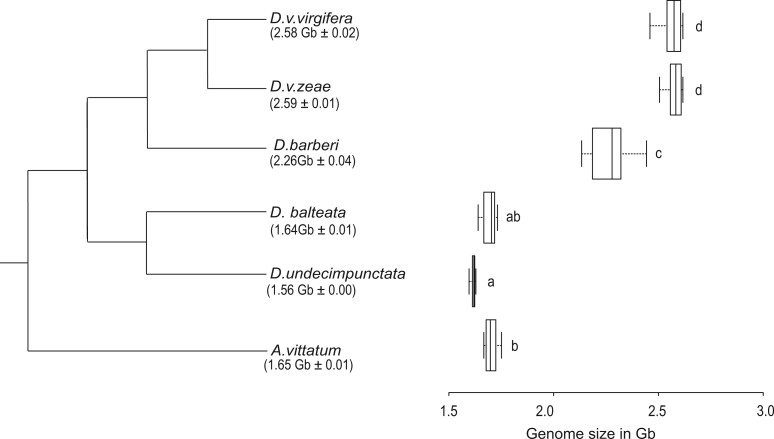
Genome size evolution within the genus *Diabrotica*. Phylogeny of *Diabrotica* and outgroup *Acalymma vittatum* is based on Eben and Espinosa de los Monteros (2013). Letters a–d indicate groups with no significant difference in mean 1C-value (Tukey HSD;α = 0.05).

All genome sizes were estimated using flow cytometry. The genome sizes of *D. v. zeae*, *D. v. virgifera*, and *D. barberi* from the virgifera group were estimated to be 2.59 Gb _**±**_ 0.01, 2.58 Gb _**±**_ 0.02, and 2.26 Gb _**±**_ 0.04, respectively. The genomes of *D. balteata* and *D.* *undecimpunctata* from the fucata group were estimated at 1.64 Gb _**±**_ 0.01 and 1.56 Gb _**±**_ 0.00. The outgroup species *A. vittatum* genome size was estimated to be 1.65 Gb _**±**_ 0.01. An ANOVA showed a significant difference *[F*_(5, 42)_ = 597, *P*** ***<*** **0.001] in the genome sizes of the species under study. A subsequent Tukey HSD test showed that there were no significant differences in genome size between *D. v. virgifera* and *D. v. zeae*, between *D. balteata* and *D.* *undecimpunctata*, or between *D. balteata* and *A. vittatum*. The estimated genome size for each species with their phylogenetic relationships is shown in Fig. 1.

### Repeat content analysis

The repeatomes of *D.* *v.* *virgifera, D. barberi*, and *D.* *undecimpunctata* comprised 72.4%, 70.3%, and 52.7% of their genomes, respectively. The repeat content obtained via Repeat Modeler for the draft *D. v. virgifera* genome was 57.4%. As the assembly of the *D.* *v. virgifera* genome was based on short-reads, TEs were expected to be under-represented because of the difficulty of assembling individual copies. TE-rich large genomes are difficult to assemble and often end up with high levels of fragmentation around repetitive regions leading to underestimation of TE content (Green 2002).

**Fig. 2.**
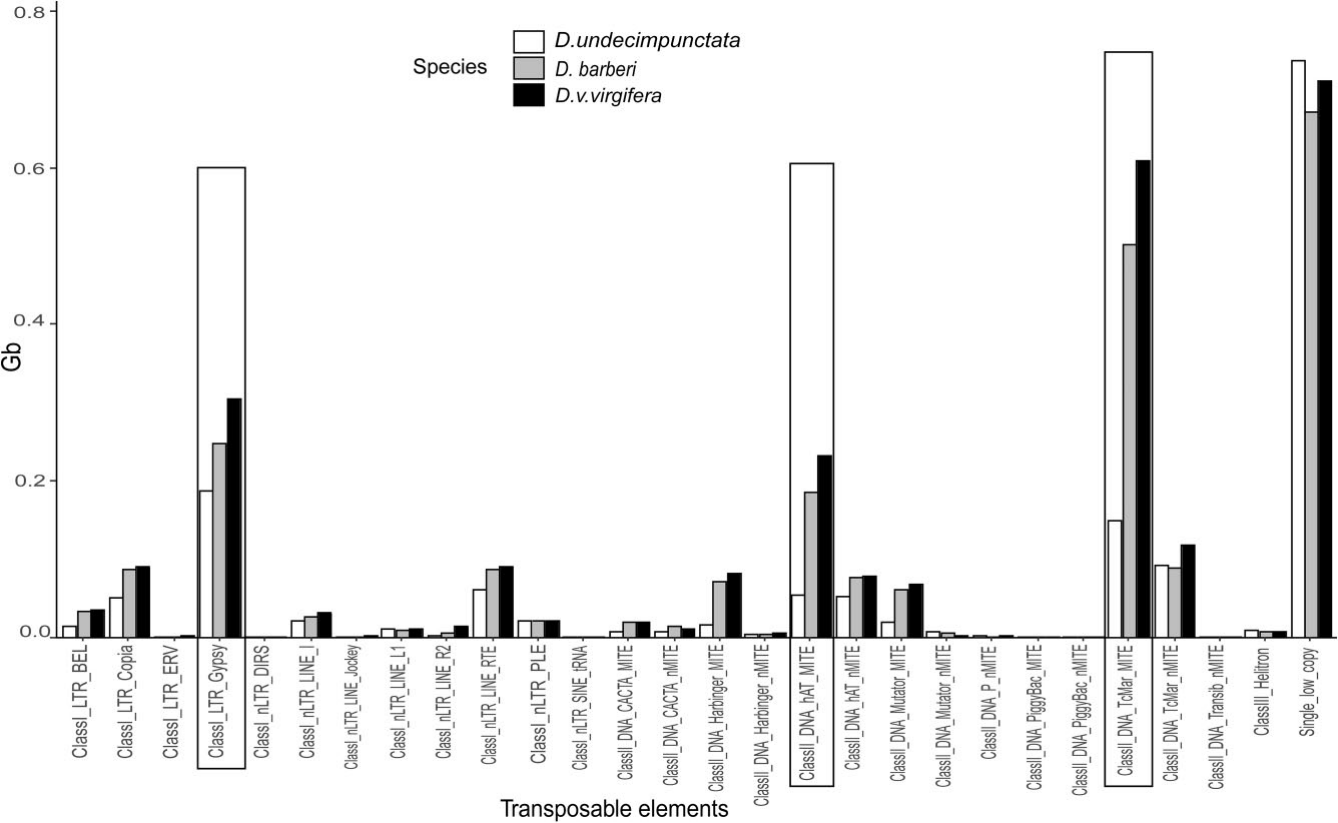
Predicted transposable elements (in Gb) in the genome of 3 species of *Diabrotica*. The boxed transposable elements are the top 3 highest contributors to the genome size variation between the groups of fucata and virgifera.

To further investigate the classes of repeat families that contributed to the genome size variation between the 2 groups of *Diabrotica*, the DeepTE annotations were coupled with the dnaPipeTE abundance quantification to estimate the abundance of different repeat elements in the genomes of *D. v. virgifera, D.* *barberi*, and *D.* *undecimpunctata*. The TEs that accounted for most of the difference in the genome size of the 2 groups were annotated as class II DNA Tc1-mariner Miniature-repeat Transposable Elements (MITEs) (ClassII_DNA_TcMar_MITE) and class II DNA hAT MITE (ClassII_DNA_hAT_MITE) of TEs and class I long terminal repeat (LTR) Gypsy (ClassI_LTR_Gypsy) TEs and is shown in Fig. 2. The *D. v. virgifera* genome contained a large amount of ClassII_DNA_TcMar_MITE(0.61 Gb), ClassII_DNA_hAT_MITE (0.23 Gb), and ClassI_LTR_Gypsy(0.31 Gb) (Fig. 2). Similarly, *D. barberi* also had a high amount of ClassII_DNA_TcMar_MITE(0.50 Gb), ClassII_DNA_hAT_MITE (0.19 Gb), and ClassI_LTR_Gypsy(0.25 Gb) (Fig. 2). The *D. undecimpunctata* had a lower amount of ClassII_DNA_TcMar_MITE(0.15 Gb), ClassII_DNA_hAT_MITE (0.06 Gb), and ClassI_LTR_Gypsy(0.19 Gb) (Fig. 2). TEs from other repeat families such as nLTRS, helitrons, and others from class I and class II DNA elements were not as prominent as those mentioned above. Single-low copy sequences representing the nonrepetitive portion of the genomes of *D. v. virgifera*, *D.* *barberi*, and *D.* *undecimpunctata* totaled 0.73 Gb, 0.68 Gb, and 0.75 Gb, respectively (Fig. 2).

## Discussion

*Diabrotica* is the most diverse genus within the subtribe Diabroticina and includes 354 species native to America. Only species from the fucata and virgifera groups occur in the United States (Krysan 1986), while species from the signifera group are endemic to South America. The signifera group species are not of economic importance, and their biology is also mostly unknown (Clark et al. 2001) and so the group is understudied. The economically significant pest species in this genus either belong to the virgifera group or to the fucata group, justifying the need to study them comprehensively. The expansion and adaptability of the pest species generates a sense of urgency to study them.

Our results demonstrated that the genome size of virgifera group species are approximately 1 Gb larger than that of the fucata group and *Acalymma* species. The genome size for *D. v. virgifera* obtained in this study and in a previous study (Coates et al. 2012) are consistent. Our genome size results, when coupled with the species’ phylogenetic relationship, indicated that an expansion in genome size occurred in the common ancestor of the virgifera group leading to *D. barberi, D. v. virgifera*, and *D. v. zeae*. There was also a significant difference in the genome sizes of *D. barberi* and *D. virgifera subsp.* suggesting a possible further expansion of the genome size in the common ancestor of *D. v. virgifera* and *D. v. zeae*.

Repeat elements of species from fucata and virgifera were studied to understand the basis of genome size expansion in *Diabrotica.* Differences in the total amount of repetitive DNA accounted almost entirely for the differences in genome size between the 3 species that were studied. The total amounts of low copy number DNA (which includes most genes) were very similar, only differing by a few 10 s of Mb. These results strongly support the hypothesis of TE proliferation as the driver of genome expansion in the virgifera group. Our data do not support a role for genome duplication, as this mechanism would also produce substantial differences in the quantity of low copy number DNA.

The genomes of the virgifera and fucata group of *Diabrotica* species contained many common TEs, but the abundance of 3 TE families differed substantially and accounted for approximately 74% of the difference in genome size between the 2 groups and also 66% of the difference within the virgifera group between *D.* *barberi* and *D. virgifera subsp*. The MITE-like Tc1-mariner and hAT elements and LTR Gypsy retroelements were more abundant in the virgifera group (45% in *D. virgifera*, 42% in *D.* *barberi*). Miniature-repeat transposable elements (MITEs) are short AT-rich (<0.5 kb) derivatives of DNA elements whose internal sequence lacks an open reading frame (Lu et al. 2012), contain conserved terminal inverted repeats, are flanked by target site duplications, and are closely associated with euchromatic genes (Kuang et al. 2009). Gypsy elements are one of the most abundant classes of the long terminal repeat (LTR)-retrotransposons superfamily with large numbers of copies found in almost all the plants, animals, and fungi tested (Thomas-Bulle et al. 2018). Generally, class I TEs have been reported to be in high abundance in insect genomes, such as *T. castaneum* (Wang et al. 2008), *Drosophila* (Clark et al. 2007), and *Bombyx mori* (Osanai-Futahashi et al. 2008) in comparison to class II elements. However, in *Diabrotica*, we discovered an abundance of class II elements and, to some extent, class I elements.

Genome size varies enormously among eukaryote species (Hidalgo et al. 2017), including beetles in the family Chrysomelidae (Petitpierre et al. 1993; Hanrahan and Johnston 2011). Some have speculated that variation in genome size *per se* is related to variation in phenotypic traits in insects. Correlations have been reported between genome size and traits, including body size (Ferrari and Rai 1989; Finston et al. 1995; Palmer and Petitpierre 1996; Palmer et al. 2003), development rate (Carreras et al. 1991; Gregory et al. 2003), and indeed, host plant range (Matsubayashi and Ohshima 2015; Calatayud et al. 2016; Zhang et al. 2019). However, in many of these examples, different studies have produced contradictory results, with both positive and negative correlations for the same trait. In the case of our data, there was no relationship between host plant range and genome size. We found the cucurbit-specialist *Acalymma* had a similar-sized genome to generalists in the fucata group of *Diabrotica*, whereas the other specialist species in the study (virgifera group *Diabrotica*) had substantially larger genomes. The data from our study support the view that correlations between genome size and phenotypic traits are generally coincidental.

Our data showed that the increased genome size in the virgifera group of *Diabrotica* species was the result of the proliferation of a few TE families. Comparative genomic studies in insects have revealed that repeat elements can make large contributions to genome size variation. Variation in TE abundance can be seen both within and among species (Lynch 2007). Honeybees, with a genome size of 230 Mb, show very few repeat elements, representing a case of TE extinction (Weinstock et al. 2006). Similarly, the small genome of *Belgica antarctica*, the Antarctic midge (99 Mb), is also due to the reduction of repeats in the genome (Kelley et al. 2014). There are also cases of TE proliferations, such as in *Locusta migratoria*, that led to its large genome size of 6.5 Gb (Wang et al. 2014). Cases of increase in genome size as a consequence of TEs have also been reported in wood white (Leptidea) butterflies and North American fireflies (Lampyridae) (Lower et al. 2017; Talla et al. 2017). Overall, it appears that genome size in insects is fairly plastic and largely driven by the loss and gain of TEs.

It is likely that the proliferation of TEs responsible for the increase in genome size observed in our study occurred sometime after the divergence of the ancestors of the virgifera and fucata groups 30 million years ago, but before the radiation of the virgifera group species, around 17 million years ago (Eben and Espinosa de los Monteros 2013). Host genomes have several mechanisms to suppress TE expression and mobility (Bourque et al. 2018), including epigenetic silencing through histone modifications or DNA methylation, targeted mutagenesis, small RNA interference, as well as sequence-specific repressors such as the recently profiled KRAB zinc-finger proteins (Fouché et al. 2020; Maupetit-Mehouas and Vaury 2020). At the same time, some TEs have evolved regulatory sequences controlling their own copy number to autonomously replicate in the genome (Lohe and Hartl 1996; Saha et al. 2015). TE derepression is triggered by environmental stimuli, in particular stress (Bundo et al. 2014; Voronova et al. 2014; Fouché et al. 2020), impacting transcription levels and increasing transpositional activity (Dubin et al. 2018). In addition, there are other factors influencing TE mobilization, for example, demethylation and the removal of repressive histone marks during epigenetic reprogramming stages (Russell and LaMarre 2018). The factors that led to derepression and subsequent proliferation of TEs in the ancestor of the virgifera group of species are unknown.

Although gross variation in genome size, driven by gain and loss of TEs, does not correlate with phenotypic adaptation, mutations associated with specific TE insertions or excisions can be adaptive. Mostly, TE insertions are presumed to be deleterious or neutral, but some have been shown to be selectively advantageous. There are several studies showing that TE-mediated insertions have led to insecticide resistance. In pink bollworm *Pectinophora gossypiella*, a major pest of cotton (Rostant et al. 2012), several independent TE insertions in the *PgCad1* gene conferred resistance to *Bt* Cry1Ac toxin (Fabrick et al. 2011; Wang et al. 2019). Cases of resistance to *Bacillus thuringiensis* (Bt) toxins have also been reported in *Heliothis virescens*, which is caused by disruption of a cadherin-superfamily gene by TE insertion (Gahan 2001). TE insertions in xenobiotic metabolism-related genes such as those encoding cytochrome P450 monooxygenases and glutathione *S*-transferases in *Helicoverpa armigera* are the causes of resistance to insecticides (Klai et al. 2020). Another example demonstrating that TEs can produce adaptive mutations has been reported in Drosophila *melanogaster* (Rostant et al. 2012; Gilbert et al. 2021). An increased resistance to dichlorodiphenyltrichloroethane (DDT) in *D. melanogaster* has been reported due to Cyp6g1 upregulation caused by insertion of the *Accord* transposon in the 5_**′**_ regulatory region of the Cyp6g1 gene (Chung et al. 2007). Similarly, TE insertion, which truncates the *CHKov1* gene in *D. melanogaster*, confers resistance toward organophosphate (Aminetzach 2005).

The degree to which mutations caused by the proliferation of TEs in the virgifera group of *Diabrotica* contributed directly to the evolution of the group is unknown. Tackling this question will require a comparative evolutionary genomic analysis of the *Diabrotica* genus. To date, significant genome sequence data are only available for 1 species, *D. v. virgifera*. However, the USDA-ARS Ag100Pest initiative aims to sequence additional *Diabrotica* genomes. The information on the evolution of size and repeat content of *Diabrotica* genomes presented in this study will help to inform the optimum strategies for sequencing additional genomes for the genus and, potentially, Diabroticite genomes more generally.

## Data availability

Nucleic acid sequencing data are available from the NCBI Sequence Read Archive under the accession numbers provided above. All flow cytometry data are available for download from figshare: https://doi.org/10.25387/g3.16892323.
